# Changes in the prognosis of CADASIL over time: a 23-year study in 555 individuals

**DOI:** 10.1136/jnnp-2024-334823

**Published:** 2024-11-15

**Authors:** Nontapat Sukhonpanich, Fatemeh Koohi, Amy A Jolly, Hugh S Markus

**Affiliations:** 1 Stroke Research Group, Department of Clinical Neurosciences, University of Cambridge, Cambridge, Cambridgeshire, UK; 2 Department of Medicine, Faculty of Medicine Siriraj Hospital, Mahidol University, Bangkok, Thailand

**Keywords:** CADASIL, STROKE, CEREBROVASCULAR DISEASE, GENETICS

## Abstract

**Background:**

Cerebral autosomal dominant arteriopathy with subcortical infarcts and leukoencephalopathy (CADASIL) is the most common monogenic form of stroke and is associated with early-onset stroke and dementia. Whether its clinical phenotype is becoming milder with better risk factor treatments and other care improvements is unknown. In a large longitudinal CADASIL cohort, we determined whether the prognosis has changed over 23 years.

**Methods:**

Patients were identified from the Cambridge CADASIL register and the UK Familial stroke study. Change in age at stroke over the time of recruitment was determined using linear mixed-effects model, and the impact of genetic and vascular risk factors on stroke and dementia risk was further evaluated using Cox proportional hazard regression.

**Results:**

A total of 555 patients with CADASIL were recruited between 2001 and 2023. The age of stroke onset significantly increased over time (p<0.001), with the mean age of stroke onset for patients recruited before 2016 (n=265) at 46.7±9.2 years and 51.6±9.5 years for those recruited since 2016 (n=290). Patients recruited since 2016 had lower risks of both stroke (HR 0.36, 95% CI 0.26 to 0.50, p<0.001) and dementia (HR 0.43, 95% CI 0.19 to 0.99, p=0.046) after adjusting for sex, hypertension history, smoking status, epidermal growth factor-like repeat position and calendar effect.

**Conclusions:**

The clinical phenotype of CADASIL is improving. While this may be partly explained by reduced vascular risk factors such as smoking and the identification of milder cases, differences persisted after controlling for risk factors and mutation sites. These updated risk estimates should be used when counselling patients with CADASIL on prognosis.

**WHAT IS ALREADY KNOWN ON THIS TOPIC**

**WHAT THIS STUDY ADDS**

- In this large, longitudinal prospectively recruited CADASIL cohort, the age onset of stroke in patients with CADASIL increased significantly over the time of recruitment.
- Patients recruited since 2016 had lower risk of both stroke and dementia.
- Additionally, survival analysis showed that patients who stopped smoking at any time had a significantly higher probability of remaining stroke and dementia-free.

**HOW THIS STUDY MIGHT AFFECT RESEARCH, PRACTICE OR POLICY**

- This study provides updated risk estimates which can be used when counselling patients with CADASIL on prognosis.
- It emphasises the importance of treating cardiovascular risk factors in CADASIL.

## Introduction

 Cerebral autosomal dominant arteriopathy with subcortical infarcts and leukoencephalopathy (CADASIL) is the most common monogenic form of stroke, causing early-onset lacunar stroke and dementia. Early reports described a mean age of onset of stroke in the 40s and that the disease was highly penetrant, with most patients with CADASIL suffering from disability and dementia by late middle age.^1^,^5^ However, recently, the perception of many clinicians is that the disease may be becoming milder, with increasing numbers of patients remaining stroke and disability-free at later ages.

Over the last decade, in developed countries, there has been a marked decline in stroke incidence, perhaps reflecting better treatment of cardiovascular risk factors.^6 7^ For example, a study based in England noted that between 2000–2003 and 2012–2015, the age-adjusted incidence of ischaemic stroke decreased by 43%.^8^ Although CADASIL is a monogenic disorder, conventional cardiovascular risk factors alter its severity. Both smoking and hypertension have been shown to have a marked effect on the CADASIL phenotype, with stroke occurring approximately 10 years earlier in active smokers.^9 10^ It is possible that an improvement in the CADASIL phenotype may have occurred, paralleling the reduced incidence seen in sporadic stroke. It is also possible that as the disease has become increasingly recognised, milder and less penetrant cases have been diagnosed, which would result in an improvement in the CADASIL phenotype.

To determine whether there have indeed been changes in the severity of the CADASIL phenotype over time, we studied a prospectively recruited cohort of patients with CADASIL recruited over 23 years to determine whether the age of onset of first stroke and dementia had changed. We then determined whether any change was explained by alterations in risk factor profiles or other factors, including mutation location.

## Methods

### Participants and recruitment

Patients were studied from two prospective UK CADASIL disease registers: the UK CADASIL National Referral Service at Cambridge (2001–2023) and the UK Familial Small Vessel Disease (SVD) study, which prospectively recruited patients from six neurological centres in England between 2016 and 2023. A total of 555 patients with CADASIL were included in this study; data on 200 have already been reported in a previous study.^9^ The inclusion criterion was a diagnosis of CADASIL on genetic testing, with a typical cysteine-changing mutation.

All patients gave written informed consent for their data to be used for research.

### Clinical parameters

Clinical details were prospectively collected using a standardised proforma. Clinical details recorded included the presence and age at onset of stroke, migraine, psychiatric symptoms, encephalopathic episodes, seizures and dementia. Stroke was defined as a focal neurological deficit lasting more than 24 hours, accompanied by neuroradiological evidence of an ischaemic or haemorrhagic lesion. Migraine with or without aura was defined by the third edition of the International Classification of Headache Disorders (ICHD-3).^11^ History of any psychiatric symptoms was recorded. Depression was defined by a history of low mood requiring medical treatment or psychotherapy. CADASIL encephalopathy was described as an acute reversible encephalopathic episode without any other organic causes, lasting more than 24 hours and warranting a hospital admission.^12^ Epileptic seizures were only recorded if they were not related to an encephalopathic episode. Dementia was defined when it was previously diagnosed by a neurologist or psychiatrist or following the Diagnostic and Statistical Manual of Mental Disorders Text Revision, Fourth Edition (DSM-IV-TR) criteria.^13^ The onset of symptoms was defined as the age when the first event of each symptom occurred.

Cardiovascular risk factors were recorded. Hypertension was defined as an elevated systolic and/or diastolic blood pressure ≥140 and 90 mm Hg or the use of antihypertensive treatment. Hypercholesterolaemia was defined as an elevated total cholesterol level ≥5.2 mmol/L or on a lipid-lowering agent. Diabetes mellitus was defined as having previously diagnosed with diabetes, the use of an anti-diabetic drug or having a fasting plasma glucose ≥7.0 mmol/L. Smoking was categorised as current, ex and never smoking. The age at onset of risk factor was defined as the age at which the disease was diagnosed or treatment was started.

### Statistical analysis

Demographic and clinical characteristics of patients were presented as mean, SD, median and IQR for continuous variables and as frequency (%) for categorical variables. As it has been previously demonstrated that patients with mutations in epidermal growth factor-like repeat (EGFr) domain 1–6 exhibit more severe disease,^14^ mutation sites were categorised into two groups: mutations affecting EGFr domain 1–6 and those affecting 7–34.

To explore changes in age at first stroke over time, we applied a linear mixed-effects model, using age at first stroke as the dependent variable and the year of recruitment as the independent variable. The mixed-effects model specification was employed to account for likely correlations among data from multiple members of a single family and calendar time. In the analysis, adjustments were made for sex, hypertension, smoking status and EGFr domain by treating them as fixed effects. Additionally, control for the variation arising from calendar time was implemented by considering the birth cohort (10-year intervals) as a random effect. The ‘lmer’ function of the ‘lme4’ R package was used to fit the model. This analysis was not performed for age at dementia due to the small number of patients with dementia in the cohort.

We additionally divided patients into two groups of approximately equal size based on recruitment year: those recruited before 2016 and from 2016 to 2023. This cut-off was decided prior to data analysis. Differences between groups were analysed using χ^2^ test and Student’s t-test, depending on variable types. We compared age at onset of first stroke and dementia using time-to-event analysis. This allowed the inclusion of data from individuals who had not yet experienced an event (stroke or dementia) at the time of the last follow-up. Analysis was performed using Kaplan-Meier survival analysis, and survival time comparisons were made using the log-rank test. Cox proportional hazard regression was used to evaluate the impact of other independent variables on the risk of stroke and dementia. Because age from birth served as the timescale, we conducted a Cox model stratified by birth cohort (10-year intervals) to account for calendar effects. Recruitment period, sex, hypertension, smoking status and EGFr domain were considered in the model. To assess whether the changes in age at stroke and dementia were accounted by which variables, we created four different Cox models controlling for different sets of variables, including only sex (model 1), sex and mutation site (model 2), sex and vascular risk factors (model 3), and sex, mutation site and vascular risk factors (model 4). Nagelkerke’s pseudo-R^2^ was calculated to compare the variance explained by each model. A two-sided p value <0.05 was considered statistically significant, with no adjustment for multiple comparisons. Kaplan-Meier curves were created using ‘survminer’ package. All statistical analyses were carried out using the R software V.4.3.2.

## Results

### Clinical spectrum of 555 patients with CADASIL and their risk profile

Five hundred fifty-eight patients with CADASIL were recruited. All were heterozygotes except for one whose mutation was compound heterozygous and one was homozygous. These two cases and one with incomplete data were omitted, leaving 555 in the analysis. Of these, 526 (94.8%) were symptomatic, and 29 (5.2%) were asymptomatic diagnosed on predictive testing. The mean age at recruitment was 49.2±11.9 years, and the mean age at first presentation in those with symptoms was 31.7±14.4 years. All patients had cysteine-altering *NOTCH3* mutations; 433 (78.0%) were in EGFr domain 1–6. Clinical features and prevalence of cardiovascular risk factors are summarised in table 1, and risk factor profiles for each clinical feature are provided in online supplemental table 1. The distribution of age at the onset of migraine, stroke and dementia is shown in online supplemental figure 1.

**Table 1:** Demographic and clinical characteristics of patients by recruitment year

Characteristics		All cases (n=555)	Recruitment before 2016 (n=265)	Recruitment since 2016 (n=290)	P value
**Demographic data**					
Born before 1964		295 (53.2)	180 (67.9)	115 (37.9)	**<0.001**
Age at recruitment; mean±SD, years		49.2±11.9	47.8±11.7	50.5±12.0	**0.007**
Male sex; n (%)		239 (43.1)	116 (43.8)	123 (42.4)	0.812
EGFr 1–6; n (%)		433 (78.0)	226 (85.3)	207 (71.4)	**<0.001**
**Clinical features**					
Migraine; n (%)		398 (71.7)	184 (69.4)	214 (73.8)	0.296
Stroke; n (%)		251 (45.2)	135 (50.9)	116 (40.0)	**0.012**
Psychiatric symptom; n (%)		237 (42.7)	101 (38.1)	136 (46.9)	**0.045**
Encephalopathy; n (%)		55 (9.9)	26 (9.8)	29 (10.0)	1.000
Seizure; n (%)		34 (6.1)	19 (7.2)	15 (5.2)	0.422
Dementia; n (%)		54 (9.7)	29 (10.9)	25 (8.6)	0.436
**Cardiovascular risk factors**					
Hypertension; n (%)		125 (22.5)	58 (21.9)	67 (23.1)	0.810
Current smoking; n (%)		121 (21.8)	80 (30.2)	41 (14.1)	**<0.001**
Diabetes; n (%)		31 (5.6)	13 (4.9)	18 (6.2)	0.630
**Age onset of clinical features**					
Age onset of stroke; years	Mean±SD	49.0±9.6	46.7±9.2	51.6±9.5	**<0.001**
	Median (IQR)	49.0 (42.0–56.0)	48.0 (40.5–53.0)	50.5 (44.0–59.2)	
Age onset of dementia; years	Mean±SD	58.6±8.3	57.3±7.9	60.2±8.7	0.203
	Median (IQR)	58.0 (53.2–64.8)	57.0 (53.0–63.0)	59.0 (54.0–66.0)	

P value <0.05 is considered significant (highlighted in **bold**).

EGFr, epidermal growth factor-like repeat.

### Differences in CADASIL characteristics in patients recruited before and since 2016

**Figure 1:**
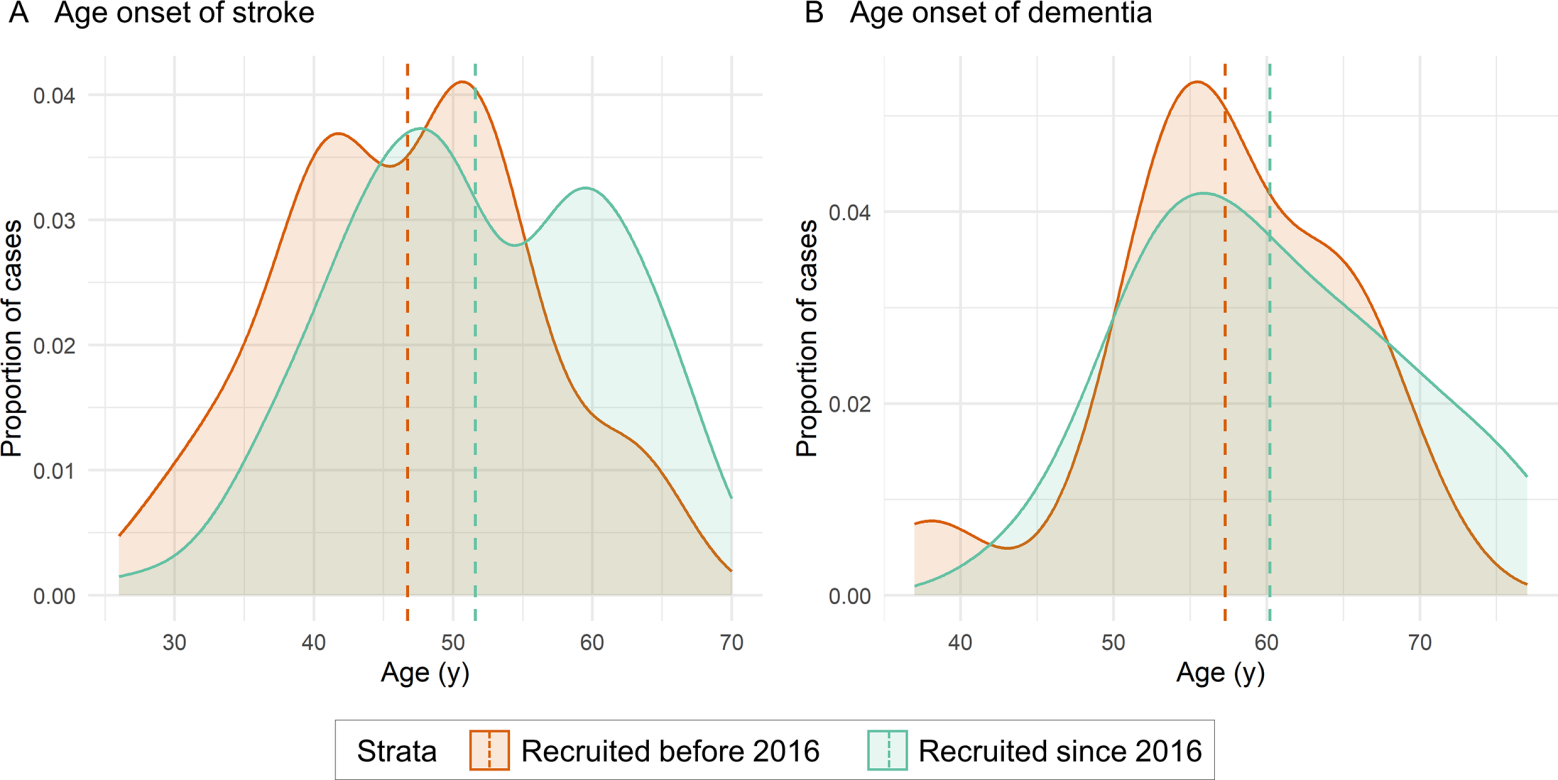
Comparison of the distribution of age at onset of stroke and dementia stratified by recruitment year before and from 2016. (A) The mean age at onset stroke was 46.7±9.2 and 51.6±9.5 years in patients recruited before and from 2016 (p<0.001), and (B) 57.3±7.9 and 60.2±8.7 years for dementia (p=0.203).

There were 265 and 290 patients with CADASIL recruited before 2016 and since 2016, respectively. Patients recruited since 2016 were more likely to be recruited at an older age (50.5±12.0 vs 47.8±11.7, p=0.007), less likely to have a history of stroke (40.0% vs 50.9%, p=0.012), more likely to have a history of psychiatric symptoms (49.6% vs 38.1%, p=0.045), less likely to smoke at the time of stroke (14.1% vs 30.2%, p<0.001) and less likely to have a mutation in EGFr 1–6 (71.4% vs 85.3%, p<0.001). The mean age at onset of stroke was 46.7±9.2 and 51.6±9.5 years, respectively, in patients recruited before and since 2016 (p<0.001), and 57.3±7.9 and 60.2±8.7 years, respectively, for dementia (p=0.203) (figure 1). Differences in baseline characteristics, clinical features and other risk factor profiles at stroke are summarised in table 1.

### Stroke

Two hundred fifty-one (45.2%) individuals had a history of stroke, of whom 246 (98.0%) experienced a lacunar ischaemic stroke and 10 (4.0%) experienced intracerebral haemorrhage; 4 (1.6%) experienced both stroke subtypes. The mean age at stroke onset was 49.0±9.6 years, and stroke was the first presenting feature in 75 (14.3%) patients.

**Figure 2:**
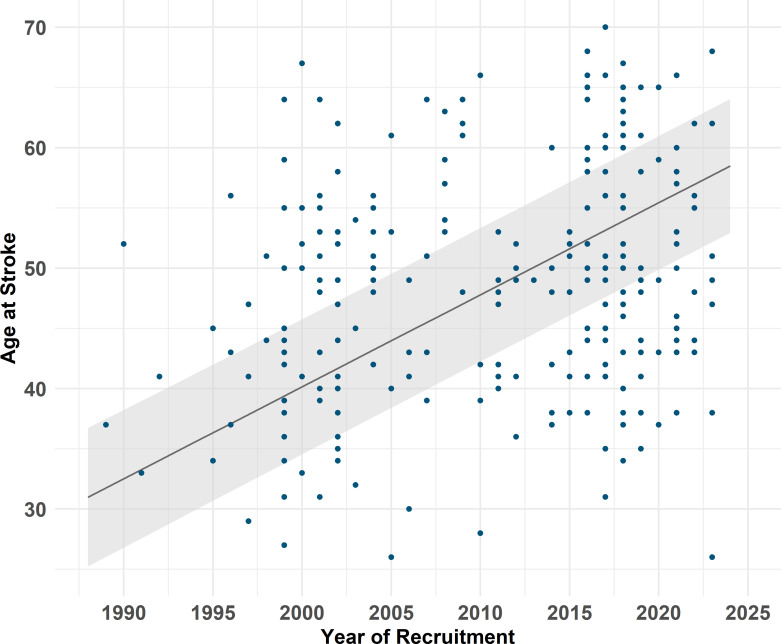
Predicted means of age at stroke onset over years of recruitment to the clinic. The points show the age at stroke for each patient.

**Figure 3:**
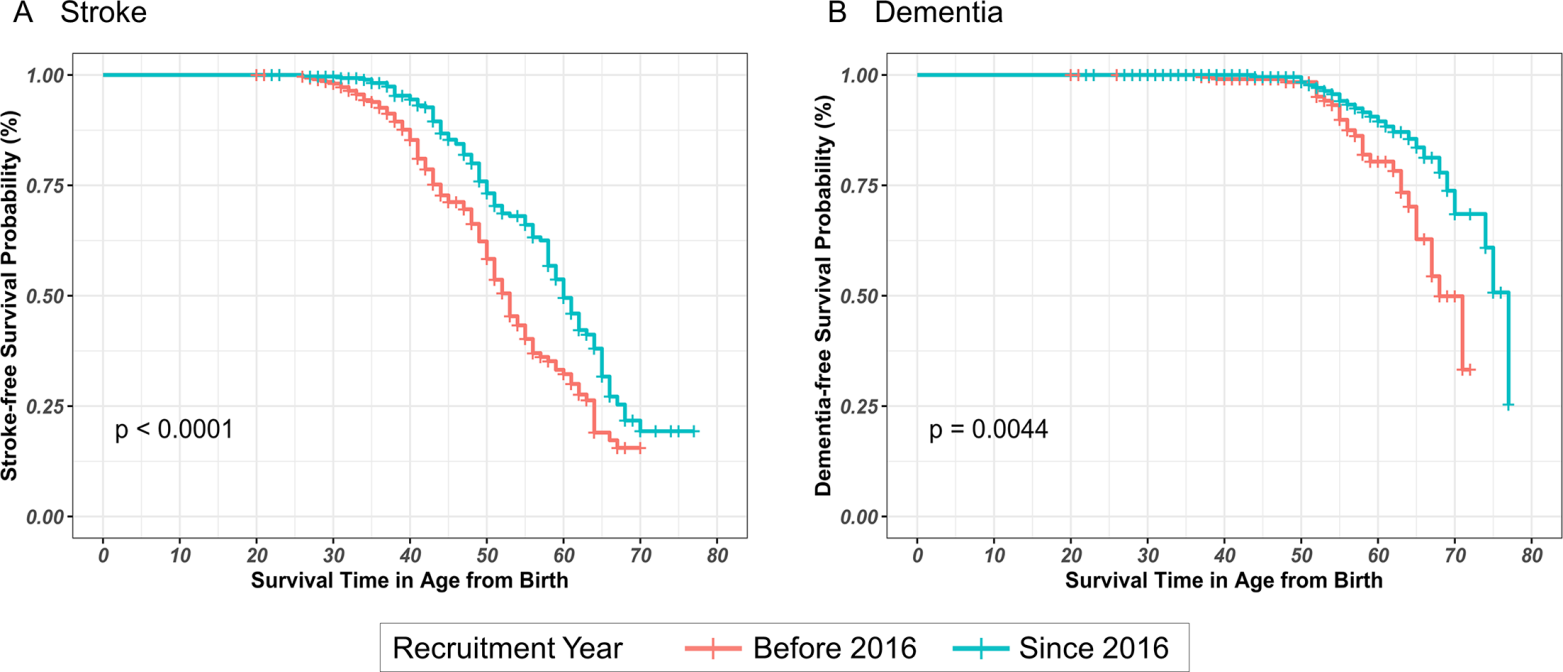
Kaplan-Meier survival estimates. Comparison of (A) stroke-free and (B) dementia-free survival in patients recruited since 2016 and those recruited before 2016. The median stroke-free survival time was 60.0 years (95% CI 58.0 to 64.0) among those recruited since 2016 and 53.0 years (95% CI 51.0 to 55.0) among those recruited before 2016. Median dementia-free survival time was 77.0 years (95% CI 74.0 to 77.0) in those recruited since 2016 and 68.0 years (95% CI 65.0 to 72.0).

In those with stroke, there was a gradual increase in age at first stroke between 2001 and 2023 (figure 2), which was significant after controlling for birth cohort effect, sex and EGFr position (p<0.001) and remained significant after additionally controlling for hypertension and smoking status (p<0.001). The only risk factor associated with age at stroke onset was current smoking (p=0.009), which decreased age at stroke onset (online supplemental table 2 and figure 2,3).

Figure 3A shows the stroke-free survival probability of patients recruited since 2016 compared with those recruited before 2016. Kaplan-Meier analysis indicated that age of onset of first stroke was significantly higher in those recruited since 2016 (p <0.001). The median stroke-free survival time was 60.0 years (95% CI 58.0 to 64.0) in those recruited since 2016 and 53.0 years (95% CI 51.0 to 55.0) in those recruited before 2016. When further stratifying with smoking status, the difference in survival probability still remained significant in all groups (p<0.001). The median survival time was 55.0 (95% CI 53.0 to 61.0) and 61.0 (95% CI 59.0 to 65.0) years in never or former smokers recruited before and after 2016 and was 49.0 (95% CI 47.0 to 51.0) and 51.0 (95% CI 47.0 to 65.0) in current smokers recruited before and after 2016, respectively (online supplemental figure 4).

The results of the Cox proportional hazard model, stratifying by birth cohort (10-year intervals), are presented in table 2. Patients recruited after 2016 had a lower stroke risk when controlling for sex (model 1: HR 0.28, 95% CI 0.20 to 0.38, p<0.001); after further adjusting for mutation site (model 2: HR 0.30; 95% CI 0.22 to 0.42; p<0.001); vascular risk factors (model 3: HR 0.33; 95% CI 0.24 to 0.45; p<0.001), and mutation site and vascular risk factors (model 4: HR 0.36; 95% CI 0.26 to 0.50; p<0.001). In the fully adjusted model 4, current smoking (HR 2.09; 95% CI 1.56 to 2.82; p<0.001), male sex (HR 1.61; 95% CI 1.25 to 2.06; p<0.001) and EGFr domain 1–6 (HR 1.64; 95% CI 1.18 to 1.30; p=0.004) were predictors of younger age at first stroke. Regarding this result, we further stratified patients with the EGFr group in the Kaplan-Meier analysis, and the difference in survival probability was still significantly different in all groups (p<0.001) (online supplemental figure 5).

**Table 2:** Multivariable Cox proportional hazard model stratifying by birth cohort for stroke

Risk factor*	Model 1		Model 2		Model 3		Model 4	
	HR (95% CI)	P value	HR (95% CI)	P value	HR (95% CI)	P value	HR (95% CI)	P value
Recruited since 2016	0.28 (0.20 to 0.38)	**<0.001**	0.30 (0.22 to 0.42)	**<0.001**	0.33 (0.24 to 0.45)	**<0.001**	0.36 (0.26 to 0.50)	**<0.001**
Male sex	1.58 (1.23 to 2.02)	**<0.001**	1.58 (1.23 to 2.03)	**<0.001**	1.62 (1.26 to 2.08)	**<0.001**	1.61 (1.25 to 2.06)	**<0.001**
EGFr 1–6	–	–	1.61 (1.16 to 2.23)	**0.005**	–	–	1.64 (1.18 to 2.30)	**0.004**
Hypertension	–	–	–	–	1.34 (1.01 to 1.77)	**0.043**	1.29 (0.98 to 1.71)	0.073
Current smoking	–	–	–	–	2.04 (1.52 to 2.74)	**<0.001**	2.09 (1.56 to 2.82)	**<0.001**
	Pseudo-R^2^=0.132		Pseudo-R^2^=0.146		Pseudo-R^2^=0.170		Pseudo-R^2^ (no smoking)=0.150 Pseudo-R^2^ (with smoking)=0.184	

P value <0.05 is considered significant (highlighted in **bold**).

* The presence of risk factor at stroke onset was used.

EGFr, epidermal growth factor-like repeat.

### Dementia

Fifty-four (9.7%) individuals had a diagnosis of dementia with a mean age at onset of 58.6±8.3 years. Of these, 35 (64.8%) had a history of stroke prior to dementia diagnosis.

Figure 3B shows the dementia-free survival probability of patients recruited before and since 2016. On Kaplan-Meier estimates, those recruited since 2016 had a significantly older age at dementia onset (p=0.004). Median dementia-free survival time was 77.0 years (95% CI 74.0 to 77.0) in those recruited since 2016 and 68.0 years (95% CI 65.0 to 72.0) in the other group. When further stratifying with smoking status, the difference in survival probability still remained significant in all groups (p<0.001). The median survival time was 71.0 (95% CI 67.0 to 72.0) and 77 (95% CI 74.0 to 77.0) years in never or former smokers recruited before and after 2016, and was 64.0 (95% CI 62.0 to 64.0) and 65.0 (95% CI 60.0 to 68.0) in current smokers recruited before and after 2016, respectively (online supplemental figure 6).

The results of the Cox proportional hazard model, stratifying by birth cohort (10-year intervals), are presented in table 3. The risk of dementia was lower in those recruited since 2016 after controlling for sex (model 1: HR 0.38, 95% CI 0.17 to 0.85, p=0.019); after further adjusting for EGFr site (model 2: HR 0.41; 95% CI 0.18 to 0.93; p=0.032); vascular risk factors (model 3: HR 0.39; 95% CI 0.17 to 0.87; p=0.021) and mutation site and vascular risk factors (model 4: HR 0.43; 95% CI 0.19 to 0.99; p=0.046). In the fully adjusted model 4, predictors of dementia were male sex (HR 2.65; 95% CI 1.51 to 4.63; p<0.001) and current smoking (HR 3.67; 95% CI 1.82 to 7.48; p<0.001).

**Table 3:** Multivariable Cox proportional hazard model stratifying by birth cohort for dementia

Risk factor*	Model 1		Model 2		Model 3		Model 4	
	HR (95% CI)	P value	HR (95% CI)	P value	HR (95% CI)	P value	HR (95% CI)	P value
Recruited since 2016	0.38 (0.17 to 0.85)	**0.019**	0.41 (0.18 to 0.93)	**0.032**	0.39 (0.17 to 0.87)	**0.021**	0.43 (0.19 to 0.99)	**0.046**
Male sex	2.36 (1.36 to 4.10)	**0.002**	2.36 (1.36 to 4.09)	**0.002**	2.62 (1.50 to 4.58)	**<0.001**	2.65 (1.51 to 4.63)	**<0.001**
EGFr 1–6	–	–	1.48 (0.75 to 2.91)	0.262	–	–	1.58 (0.79 to 3.16)	0.198
Hypertension	–	–	–	–	0.75 (0.41 to 1.38)	0.358	0.78 (0.42 to 1.43)	0.419
Current smoking	–	–	–	–	3.47 (1.73 to 6.94)	**<0.001**	3.69 (1.82 to 7.48)	**<0.001**
	Pseudo-R^2^=0.054		Pseudo-R^2^=0.059		Pseudo-R^2^=0.097		Pseudo-R^2^ (no smoking)=0.065 Pseudo-R^2^ (with smoking)=0.103	

P value <0.05 is considered significant (highlighted in **bold**).

* The presence of risk factor at dementia onset was used.

EGFr, epidermal growth factor-like repeat.

## Discussion

Our results, from a cohort of CADASIL subjects prospectively recruited over a 23-year period, show a marked improvement in the clinical phenotype with increasing age at the onset of the first stroke and dementia. We have demonstrated that a number of factors, including smoking and the location of the mutation, affect the disease severity, but neither fully explained the reduction in disease severity observed in our cohort.

In longitudinal time trend analysis, we demonstrated that the age onset of first stroke in subjects suffering from stroke had increased progressively and significantly. Including data from the whole cohort using a time-to-event analysis, we showed that subjects recruited since 2016 had a median stroke-free survival time 7 years later than those recruited before 2016 (60.0 years vs 53.0 years). Early studies reported the mean age of stroke in CADASIL at the 40s (41.2–46.1 years).^1 3 4^ More recent reports suggest that it may have increased to the late 40s or early 50s (49.3–52.0 years).^15^,^18^ Our data suggest that the observed age of onset is not considerably later (49.0 years) and provides useful up-to-date information for counselling on stroke risk to CADASIL mutation carriers.

We found a similar reduction in disease severity when dementia was considered, with time-to-event analysis showing the median dementia-free survival time increased to 77 years in those recruited from 2016, compared with 68 years in those recruited before 2016.

There are several explanations for this improvement in prognosis, including reduced cardiovascular risk factor exposure and diagnosis of milder cases as awareness of the disease increases. Cardiovascular risk factors have been shown to aggravate the disease phenotype. For example, smoking, hypertension and diabetes have been associated with increasing stroke risk in CADASIL,^9 19 20^ while blood pressure, even across the normal range, has been associated with increased brain atrophy.^21^ It is possible that a reduction in risk factor exposure and better treatment of risk factors could explain part of the improvement in disease severity. Studies in the UK have showed that control of some cardiovascular risk factors has improved over this period. The proportion of adults with untreated hypertension (systolic blood pressure ≥140 mm Hg and/or diastolic ≥90 mm Hg, not currently taking medication for blood pressure) decreased from 2003 to 2019 for both men (20% to 14%) and women (16% to 11%).^22^ However, the only risk factor we found to have a significant reduction during the duration of this study was smoking. Although there was an association of smoking with age of onset of both stroke and dementia, this explained little of the change in age of onset of both stroke and dementia over time. We approximated the variance explained by smoking to be 3–4% (0.034 for stroke and 0.038 for dementia) by comparing the difference in pseudo-R^2^ values between models with and without smoking. Interestingly, we found that despite smoking in the past, patients recruited at any time who stopped smoking experienced stroke and dementia at a significantly older age, emphasising the importance of smoking cessation at any time.

An alternative explanation is that milder cases have been diagnosed as disease awareness has increased. Early genetic testing for CADASIL tended to screen only those exons most commonly affected and exclude more distal exons.^23^ More recent data have shown that mutations in the proximal EGFr 1–6 are associated with an earlier age of onset of stroke and more severe disease.^14 24^ Furthermore, analyses of sequencing databases have shown that the frequency of typical CADASIL-type mutations is much more common in the general population than expected from the epidemiological estimates of the frequency of CADASIL.^10 25 26^ Therefore, it is possible that increasing numbers of milder cases, perhaps with more distal mutations, have been diagnosed, which have accounted for the milder disease severity currently observed. Supporting this hypothesis, an increasing number of distal (EGFr 7 and beyond) mutations were recruited over time (before 2016, 14.7%; after 2016, 28.6%). However, while EGFr 1–6 was a risk factor for earlier onset of stroke, entering it in the multivariable analysis had only little effect on the strength of association between time of recruitment and age of stroke onset. Therefore, the reduction in disease severity is not fully explained by changes in the number of subjects with distal EGFr. However, we cannot exclude the diagnosis of milder cases resulting in the improvement in prognosis we have seen over time.

Hence, neither cardiovascular risk factors nor mutation sites can explain most of the change in disease severity over time, and its cause remains uncertain. Lifestyle interventions such as exercise, alcohol consumption and the Mediterranean diet, which are currently more widely known, could also play a role in the recent improvements seen in CADASIL phenotypes. Despite this, our study has an important implication in clinical practice. It provides up-to-date information with which to counsel patients with CADASIL and mutation carriers on likely prognosis and suggests this is more favourable than previously believed, especially in those who carry mutations affected more distal EGFr and those who do not smoke or have quit smoking. Most importantly, it offers strong evidence supporting lifestyle modifications, such as smoking cessation or control of vascular risk factors, which might improve the disease prognosis.

Our study has several strengths. By studying a large cohort of subjects recruited over a long time period, we were able to have the power to detect time trends. All data were collected prospectively using the same definition for disease and risk factors throughout. Using linear mixed-effects and stratified Cox regression models strengthened the validity of the findings by accounting for potential correlations and variations arising from the same families and birth cohort. However, it also has limitations. Being an observational study in nature, it is difficult to demonstrate a direct causal relationship between variables and the delay in stroke onset. We used a diagnosis of definite dementia diagnosed by a dementia expert, but not all patients have such an assessment so the number of cases may be underestimated and the date of dementia onset can be difficult to accurately define. In addition, the power of the analysis in patients with dementia was limited due to the low prevalence of dementia in the cohort. Some other factors that might affect the risk of stroke and dementia, such as socioeconomic status,^27^ were not included in the analysis. In addition, due to the low prevalence of some vascular risk factors, such as diabetes, there was not enough power to investigate its effect on age at stroke onset or stroke and dementia risk, therefore they were omitted. We did not have data on treatment of risk factors during follow-up, and an intervention was not incorporated into the analytical models, making it impossible to determine the effect of treatment on the change in CADASIL prognosis. Further prospective studies with documentation of treatment changes are needed. Lastly, these results are from the UK, and need replicating in other populations. Data from the other continents do suggest that risk factors are associated with a more severe phenotype in other populations, for example, including Taiwan.^20^ However, recent data have also reported that stroke incidence is rising in many low- and middle-income countries (LMICs), in contrast to the fall seen in many higher income countries such as the UK. This could make trends in the severity of CADASIL phenotype differ across countries, and particularly between the UK and LMICs.^7^

## Conclusion

In summary, our study has shown that the prognosis of CADASIL is improving, as evidenced by the age at first stroke and dementia is increasing over time. While this might partly be explained by the reduction of smoking in later recruited patients and the inclusion of milder cases, the association remained significant after controlling for mutation position and cardiovascular risk factors. Our study provides definitive data which can be used in counselling patients on CADASIL prognosis, although it needs replicating in other populations.

## Supplementary material

10.1136/jnnp-2024-334823 online supplemental file 1

## Data Availability

Data are available upon reasonable request.

## References

[1] Tournier-Lasserve E, Joutel A, Melki J (1993). Cerebral autosomal dominant arteriopathy with subcortical infarcts and leukoencephalopathy maps to chromosome 19q12. Nat Genet.

[2] Chabriat H, Vahedi K, Iba-Zizen MT (1995). Clinical spectrum of CADASIL: a study of 7 families. Cerebral autosomal dominant arteriopathy with subcortical infarcts and leukoencephalopathy. Lancet.

[3] Dichgans M, Mayer M, Uttner I (1998). The phenotypic spectrum of CADASIL: clinical findings in 102 cases. Ann Neurol.

[4] Desmond DW, Moroney JT, Lynch T (1999). The natural history of CADASIL: a pooled analysis of previously published cases. Stroke.

[5] Chabriat H, Joutel A, Dichgans M (2009). Cadasil. Lancet Neurol.

[6] Li L, Scott CA, Rothwell PM (2020). Trends in Stroke Incidence in High-Income Countries in the 21st Century: Population-Based Study and Systematic Review. Stroke.

[7] Feigin VL, Brainin M, Norrving B (2022). World Stroke Organization (WSO): Global Stroke Fact Sheet 2022. Int J Stroke.

[8] Wafa HA, Wolfe CDA, Rudd A (2018). Long-term trends in incidence and risk factors for ischaemic stroke subtypes: Prospective population study of the South London Stroke Register. PLoS Med.

[9] Adib-Samii P, Brice G, Martin RJ (2010). Clinical spectrum of CADASIL and the effect of cardiovascular risk factors on phenotype: study in 200 consecutively recruited individuals. Stroke.

[10] Cho BPH, Harshfield EL, Al-Thani M (2022). Association of Vascular Risk Factors and Genetic Factors With Penetrance of Variants Causing Monogenic Stroke. JAMA Neurol.

[11] (2018). Headache Classification Committee of the International Headache Society (IHS) The International Classification of Headache Disorders, 3rd edition. Cephalalgia.

[12] Drazyk AM, Tan RYY, Tay J (2019). Encephalopathy in a Large Cohort of British Cerebral Autosomal Dominant Arteriopathy With Subcortical Infarcts and Leukoencephalopathy Patients. Stroke.

[13] Segal DL Diagnostic and Statistical Manual of Mental Disorders (DSM-IV-TR).

[14] Cho BPH, Jolly AA, Nannoni S (2022). Association of *NOTCH3* Variant Position With Stroke Onset and Other Clinical Features Among Patients With CADASIL. Neurology (ECronicon).

[15] Mukai M, Mizuta I, Watanabe-Hosomi A (2020). Genotype-phenotype correlations and effect of mutation location in Japanese CADASIL patients. J Hum Genet.

[16] Min J-Y, Park S-J, Kang E-J (2022). Mutation spectrum and genotype-phenotype correlations in 157 Korean CADASIL patients: a multicenter study. Neurogenetics.

[17] Paraskevas GP, Stefanou MI, Constantinides VC (2022). CADASIL in Greece: Mutational spectrum and clinical characteristics based on a systematic review and pooled analysis of published cases. Eur J Neurol.

[18] Dupé C, Guey S, Biard L (2023). Phenotypic variability in 446 CADASIL patients: Impact of NOTCH3 gene mutation location in addition to the effects of age, sex and vascular risk factors. J Cereb Blood Flow Metab.

[19] Chabriat H, Hervé D, Duering M (2016). Predictors of Clinical Worsening in Cerebral Autosomal Dominant Arteriopathy With Subcortical Infarcts and Leukoencephalopathy: Prospective Cohort Study. Stroke.

[20] Lin H-J, Chen C-H, Su M-W (2024). Modifiable vascular risk factors contribute to stroke in 1080 *NOTCH3* R544C carriers in Taiwan Biobank. Int J Stroke.

[21] Peters N, Holtmannspötter M, Opherk C (2006). Brain volume changes in CADASIL: a serial MRI study in pure subcortical ischemic vascular disease. Neurology (ECronicon).

[22] NHS England Digital (2023). Health Survey for England, 2022 Part 2.

[23] Chabriat H, Joutel A, Tournier-Lasserve E (2020). CADASIL: yesterday, today, tomorrow. Eur J Neurol.

[24] Rutten JW, Van Eijsden BJ, Duering M (2019). The effect of NOTCH3 pathogenic variant position on CADASIL disease severity: NOTCH3 EGFr 1-6 pathogenic variant are associated with a more severe phenotype and lower survival compared with EGFr 7-34 pathogenic variant. Genet Med.

[25] Rutten JW, Dauwerse HG, Gravesteijn G (2016). Archetypal *NOTCH3* mutations frequent in public exome: implications for CADASIL. Ann Clin Transl Neurol.

[26] Hack RJ, Cerfontaine MN, Gravesteijn G (2022). Effect of *NOTCH3* EGFr Group, Sex, and Cardiovascular Risk Factors on CADASIL Clinical and Neuroimaging Outcomes. Stroke.

[27] Bray BD, Paley L, Hoffman A (2018). Socioeconomic disparities in first stroke incidence, quality of care, and survival: a nationwide registry-based cohort study of 44 million adults in England. Lancet Public Health.

