# Supplementary material for: Changes in the prognosis of CADASIL over time: a 23-year study in 555 individuals
Source: J Neurol Neurosurg Psychiatry. 2024 Nov 15;96(7):e334823. doi: 10.1136/jnnp-2024-334823 (PMC12322386; doi:10.1136/jnnp-2024-334823)
Supplement: online supplemental file 1 [file jnnp-96-7-s001.pdf]

Changes in the Prognosis of CADASIL over Time: A 23-year Study in 555 Individuals

Supplementary Data

Page 2 **Supplementary Table S1** Clinical phenotypes, baseline characteristics, and risk factor profile in 555 CADASIL patients, categorised by each feature.

Page 3 **Supplementary Table S2** Linear mixed-effects model showing an association between age at stroke and year of recruitment.

Page 4 **Supplementary Figure S1** Distribution of the age at onset of CADASIL features.

Page 5 **Supplementary Figure S2** Predicted means of age at stroke onset over years of recruitment to the clinic.

Page 6 **Supplementary Figure S3** Predicted means of age at stroke onset over years of recruitment to the clinic.

Page 7 **Supplementary Figure S4** Kaplan-Meier survival estimates. Comparison of stroke-free survival in current smokers at stroke onset and never or former smokers, stratified by recruitment year since 2016 and before 2016.

Page 8 **Supplementary Figure S5** Kaplan-Meier survival estimates. Comparison of stroke-free survival in EGFr 1-6 and 7-34, stratified by recruitment year since 2016 and before 2016.

Page 9 **Supplementary Figure S6** Kaplan-Meier survival estimates. Comparison of dementia-free survival in current smokers at dementia onset and never or former smokers, stratified by recruitment year since 2016 and before 2016.

Supplementary Table S1 Clinical phenotypes, baseline characteristics, and risk factor profile in 555 CADASIL patients, categorised by each feature.

	Migraine	Stroke	Psychiatric symptom	Encephalopathy	Seizure	Dementia	Asymptomatic
n (%)	398 (71.7)	251 (45.2)	237 (42.7)	55 (9.9)	34 (6.1)	54 (9.7)	29 (5.2)
Age at onset; mean (SD), y	28.3 (13.0)	49.0 (9.6)	38.8 (14.0)	44.2 (12.9)	47.4 (13.9)	58.6 (8.3)	38.3 (12.1)*
EGFr 1-6; n (%)	315 (79.1)	201 (80.1)	181 (76.4)	49 (89.1)	26 (76.5)	43 (79.6)	21 (72.4)
Male; n (%)	150 (37.7)	122 (48.6)	88 (37.1)	21 (38.2)	22 (64.7)	32 (59.3)	13 (44.8)
Hypertension†; n (%)	11 (2.9)	87 (34.7)	22 (9.7)	11 (20.8)	5 (14.7)	20 (37.0)	3 (10.3)
Current smoker†; n (%)	104 (26.1)	72 (28.9)	77 (32.5)	9 (16.4)	8 (23.5)	15 (27.8)	8 (27.6)
Diabetes†; n (%)	2 (0.5)	22 (8.8)	8 (3.4)	2 (3.7)	6 (17.6)	6 (11.1)	0 (0.0)

Abbreviations: EGFr, Epidermal growth factor-like repeat; SD, Standard deviation

*For asymptomatic patients, age at pre-symptomatic genetic testing was used.

†The presence of cardiovascular risk factors at the onset of symptoms was shown.

Supplementary Table S2 Linear mixed-effects model showing an association between age at stroke and year of recruitment.

Variable	Estimated coefficient	Standard error	p-value
Year of recruitment	0.767	0.057	**<0.001**
Sex (female)	1.432	0.780	0.066
EGFr 1-6	-1.233	0.998	0.217
Hypertension	-0.371	0.866	0.668
Current smoking	-2.259	0.865	**0.009**

Abbreviation: EGFr, epidermal growth factor-like repeat

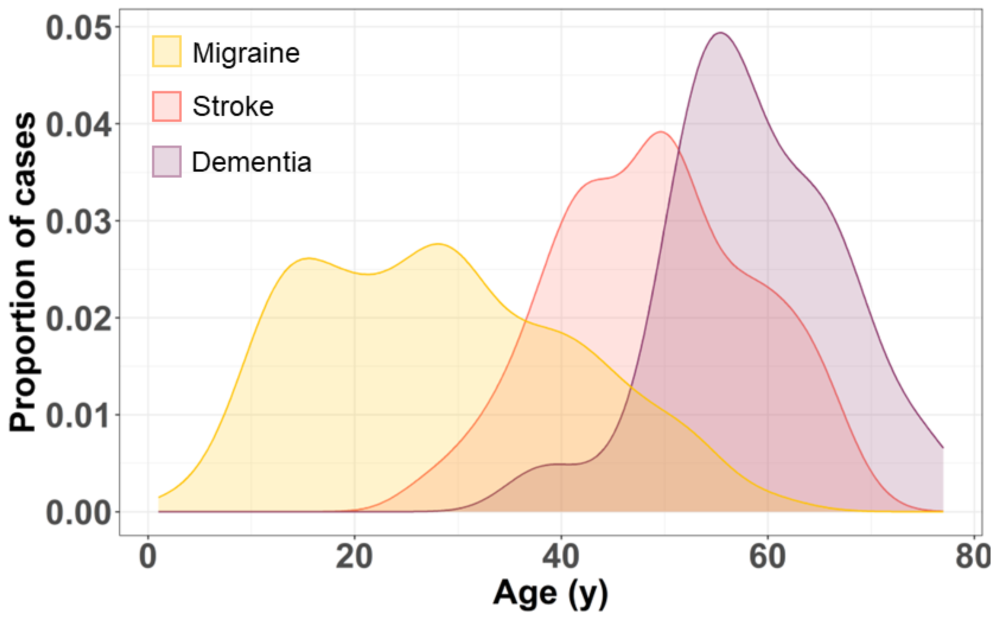

Supplementary Figure S1 Distribution of the age at onset of CADASIL features. The mean age at onset of migraine, stroke, and dementia was 28.3 ± 13.0, 49.0 ± 9.6, and 58.6 ± 8.3 years, respectively.

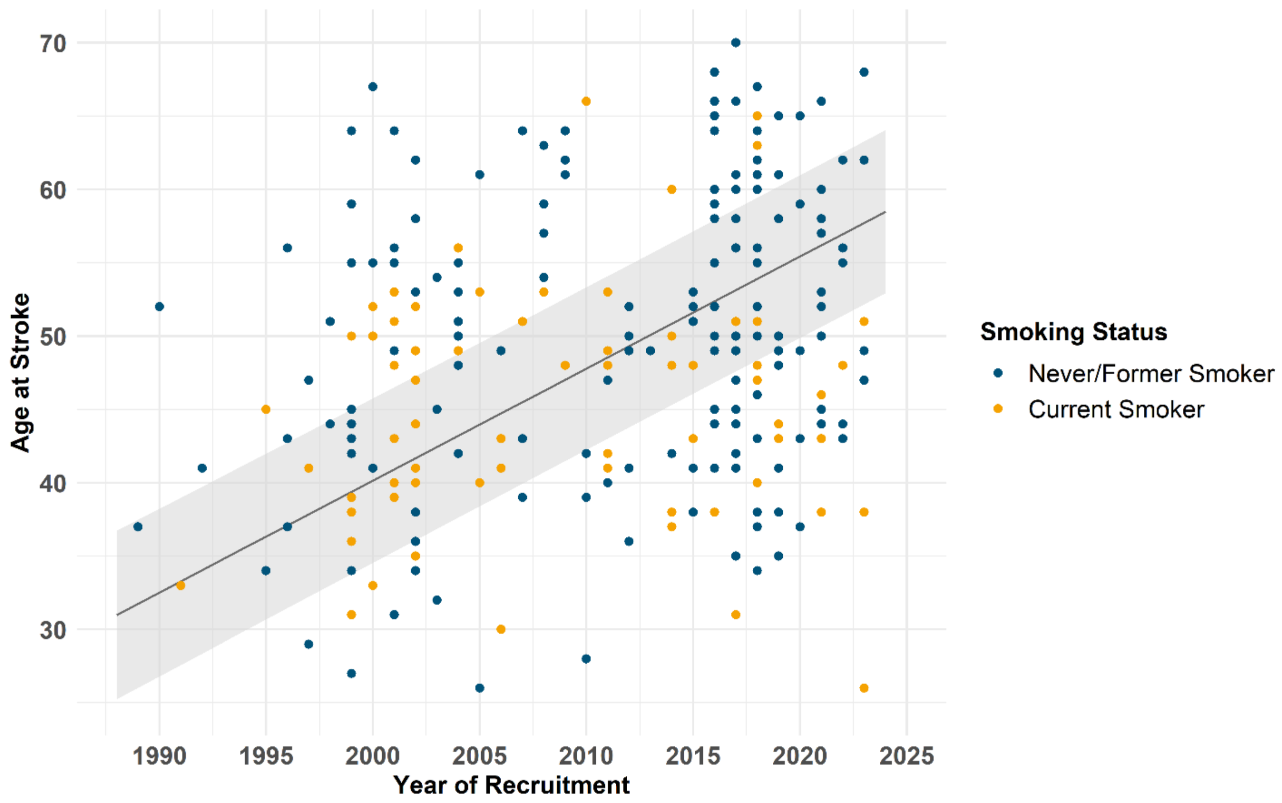

Supplementary Figure S2 Predicted means of age at stroke onset over years of recruitment to the clinic. Coloured points show the age at stroke for each patient according to their smoking status.

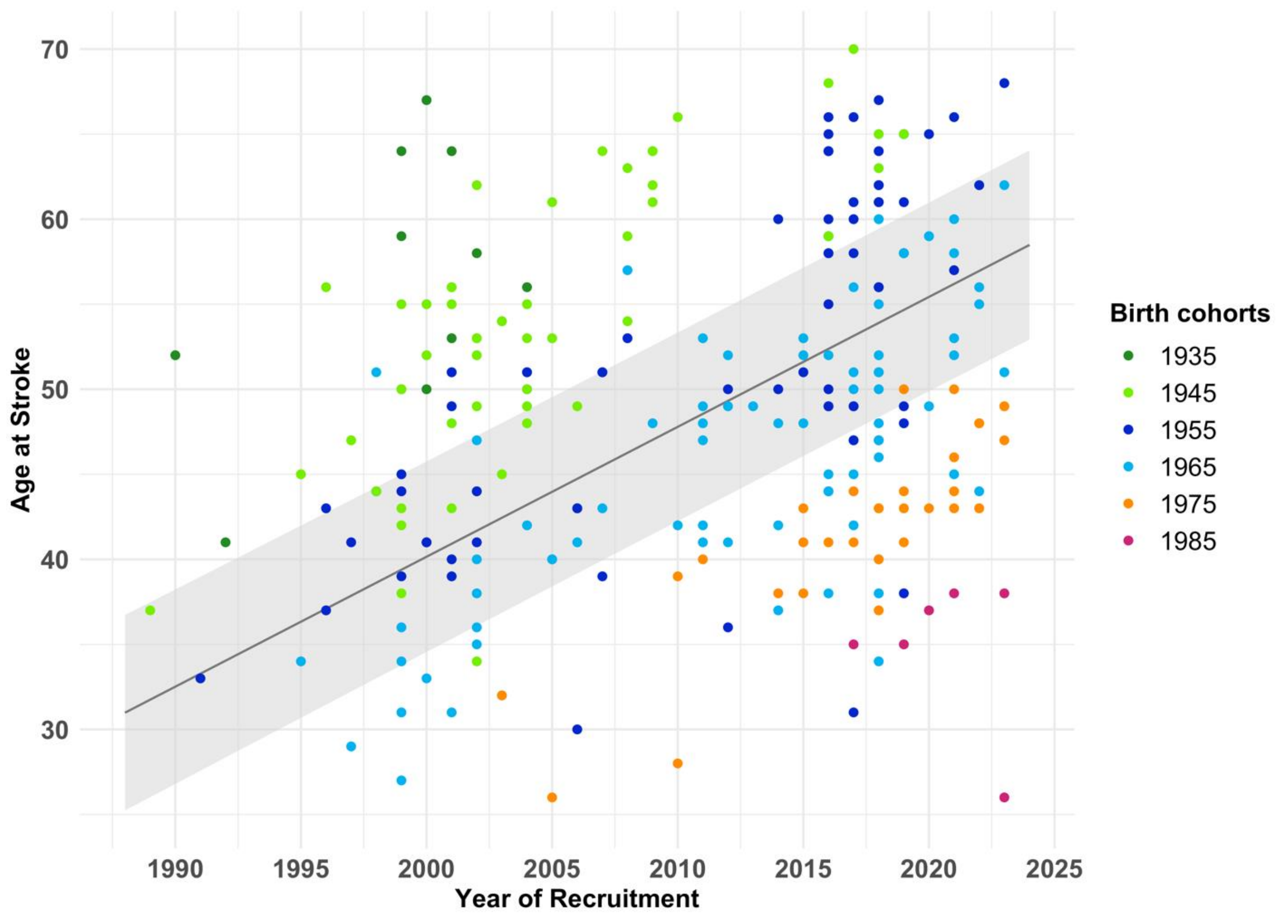

Supplementary Figure S3 Predicted means of age at stroke onset over years of recruitment to the clinic. Coloured points show the age at stroke for each patient according to their birth cohort (10-year interval: 1928-1940, 1940-1950, 1950-1960, 1960-1970, 1970-1980, 1980-1990).

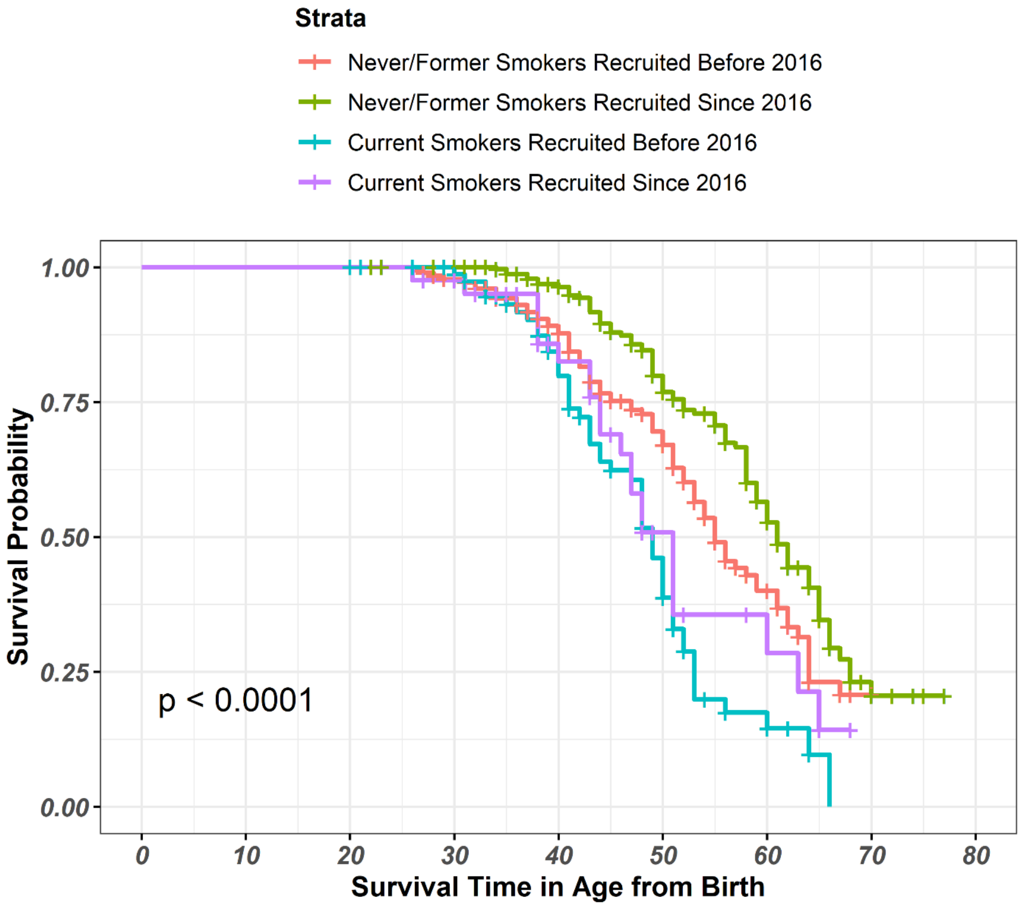

Supplementary Figure S4 Kaplan-Meier survival estimates. Comparison of stroke-free survival in current smokers at stroke onset and never or former smokers, stratified by recruitment year since 2016 and before 2016. Overall, there was a significant difference in survival probability between current and never or former smokers in all groups ($p < 0.001$). The median survival time was 55.0 (95% CI: 53.0-61.0) and 61.0 (95% CI: 59.0-65.0) years in never or former smokers recruited before and from 2016, and was 49.0 (95% CI: 47.0-51.0) and 51.0 (95% CI: 47.0-65.0) in current smokers recruited before and from 2016, respectively. In a Cox proportional hazards model including recruitment period, smoking status, and their interaction, the p-value for the interaction was 0.809, indicating that the effect of smoking status on stroke survival does not significantly differ by recruitment period.

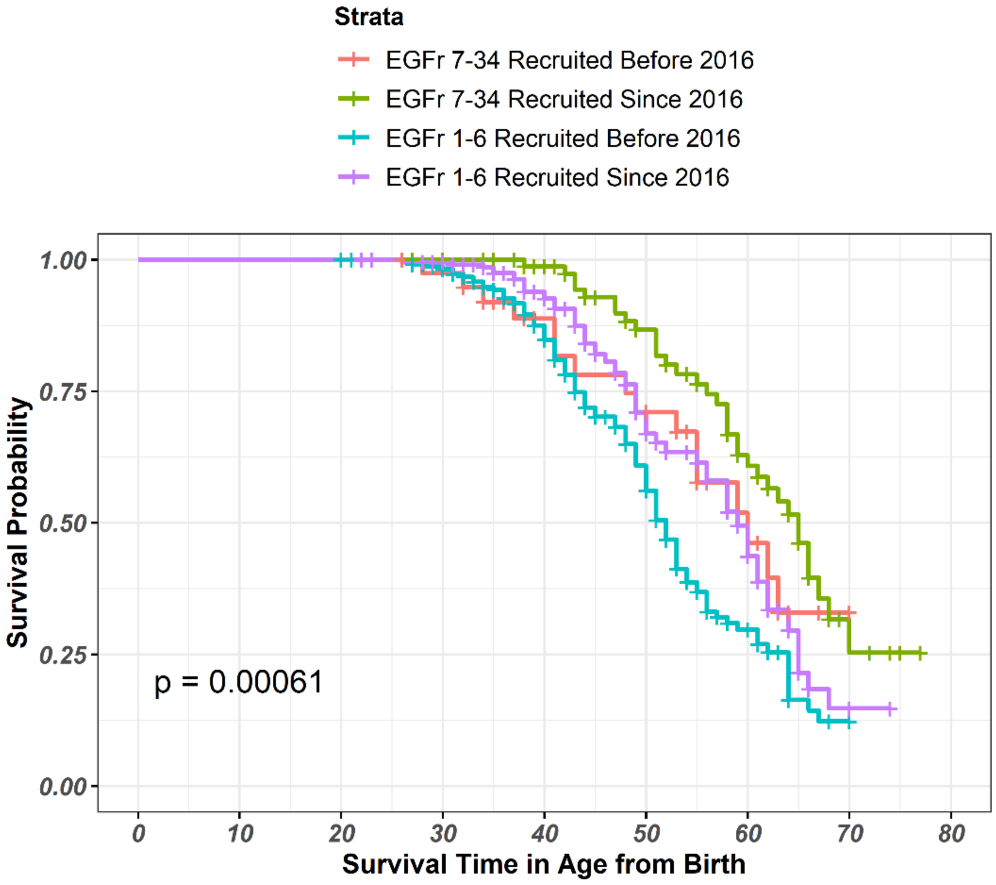

Supplementary Figure S5 Kaplan-Meier survival estimates. Comparison of stroke-free survival in patients with mutation affected EGFr 1-6 and EGFr 7-34, stratified by recruitment year since 2016 and before 2016. Overall, there was a significant difference in survival probability in all groups ($p < 0.001$). In patients who were recruited before 2016, the median survival time was 52 (95%CI 50-53) and 60 (95%CI 55-70) years in those with mutations in EGFr 1-6 and 7-34, respectively, whereas in patients who were recruited since 2016, the median survival time was 59 (95%CI 56-62) and 65 (95%CI 60-70) in those with mutations in EGFr 1-6 and 7-34, respectively.

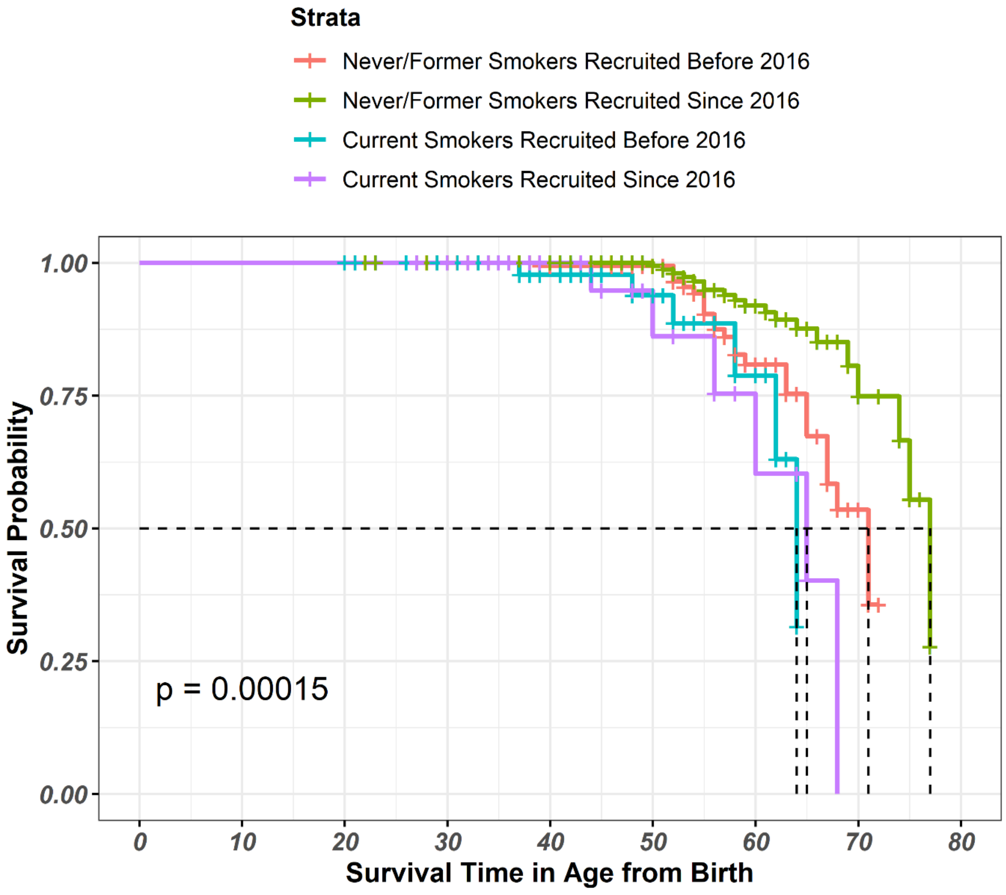

Supplementary Figure S6 Kaplan-Meier survival estimates. Comparison of dementia-free survival in current smokers at dementia onset and never or former smokers, stratified by recruitment year since 2016 and before 2016. Overall, there was a significant difference in survival probability between current and never or former smokers in all groups ($p <0.001$). The median survival time was 71.0 (95% CI: 67.0-72.0) and 77.0 (95% CI: 74.0-77.0) years in never or former smokers recruited before and from 2016, and was 64.0 (95% CI: 62.0-64.0) and 65.0 (95% CI: 60.0-68.0) in current smokers recruited before and from 2016, respectively. In a Cox proportional hazards model including recruitment period, smoking status, and their interaction, the p-value for the interaction was 0.076, indicating that the effect of smoking status on dementia survival does not significantly differ by recruitment period.
